# Outcomes of a Pilot Mobile App Just-in-Time-Adaptive-Intervention Supporting Coping Skills in Trauma Affected People with HIV

**DOI:** 10.1007/s10461-026-05032-6

**Published:** 2026-01-23

**Authors:** Rayna E. Gasik, Simone J. Skeen, Stephanie Tokarz, Katherine P. Theall, Gretchen A. Clum

**Affiliations:** 1 Department of Social, Behavioral, and Population Sciences, Celia Scott Weatherhead School of Public Health and Tropical Medicine, Tulane University, New Orleans, LA USA; 2 Connolly Alexander Institute for Data Science, Tulane University, New Orleans, LA USA; 3 Department of Epidemiology, Celia Scott Weatherhead School of Public Health and Tropical Medicine, Tulane University, New Orleans, LA USA

**Keywords:** HIV, Coping, MHealth, JITAI, Trauma-informed

## Abstract

People with HIV (PWH) experience trauma and mental health burdens at higher rates than seronegative individuals. Psychosocial interventions have shown promise for improving mental health and coping among PWH, but relatively few trauma-informed, coping-focused mobile health (mHealth) interventions exist. This pilot study evaluates the preliminary outcomes of NOLA Gem, a just-in-time adaptive intervention (JITAI)-based mHealth app designed to support real-time coping among people living with HIV (PWH) in the New Orleans area. Participants were assigned to either a control or intervention group; the intervention group received access to the NOLA Gem app, which delivered psychoeducation modules and just-in-time skill suggestions based on daily diary responses over a three-week period. Both groups completed brief daily diary surveys over the 3-week period to assess stress, emotion regulation, and coping outcomes. Analyses demonstrated positive changes in treatment compared to control, particularly in the final week. From week 1 to week 3, participants in the treatment group experienced significant reductions in maladaptive coping, rumination, and PTSD symptoms compared to the control group, along with increased feelings of control over stress at both timepoints. These effects were especially pronounced among high completers, who demonstrated greater improvements in maladaptive coping, rumination, and stress control compared to both low completers and controls. High completers also showed significant decreases in overall stress and self-reported alcohol use. These preliminary findings suggest that NOLA Gem is a promising intervention for improving coping and reducing trauma-related symptoms among PWH, particularly for those who actively engage with the app.

*Trial Registration*: ClinicalTrials.gov NCT05784714; https//clinicaltrials.gov/ct2/show/NCT05784714.

## Introduction

People living with HIV (PWH) face a disproportionate burden of mental health diagnoses, stemming from experiences of stigma, discrimination, poverty, and trauma. Some studies estimate that around 50% of PWH have experienced childhood abuse [1], and in a study of 611 individuals in the Deep South, 90% reported experiencing at least one form of trauma [2]. A global meta-analysis of 240 studies reported high rates of mental health disorders in PWH, with pooled prevalence estimates of 31% for depression, 29% for anxiety, 20% for suicidal ideation, and 20% for post-traumatic stress disorder [3]. In comparison, data from the North American AIDS Cohort revealed even higher overall rates, with 55% of the 122,896 participants diagnosed with at least one mental health condition, such as schizophrenia, anxiety, depression, or bipolar disorder, and 24% diagnosed with two or more co-occurring conditions [4]. Mental health can translate into a multitude of other health problems. For example, mental health challenges can lead to heavy drinking and substance use disorder as a coping mechanism for trauma-related stress [5]. Both substance use and mental health concerns, independently and in combination, have been shown to negatively impact the HIV care continuum, contributing to lower medication adherence [6, 7], reduced retention in care [8, 9], and poorer viral suppression outcomes [10, 11]. Conversely, engagement in mental health treatment has been shown to improve adherence and viral suppression [8, 12].

Psychosocial interventions have demonstrated positive effects for PWH experiencing mental health symptoms, including reductions in depression and anxiety, as well as improvements in coping skills [13, 14]. A meta-analysis of such interventions found that the most commonly employed approaches included traditional talk therapies (e.g., Cognitive Behavioral Therapy and Dialectical Behavior Therapy), mindfulness-based practices, and psychoeducation [13]. Reviews examining the intersection of trauma, mental health interventions, and HIV suggest that adaptive coping may play a particularly important role in influencing HIV-related outcomes [1, 15].

Living in the Face of Trauma (LIFT) is one example of a coping based intervention, grounded in Lazarus and Folkman’s coping framework [16]. LIFT is a 15-session HIV and trauma coping group intervention which guides participants in identifying and appraising stressors as changeable or unchangeable and teaches corresponding adaptive coping strategies, such as problem-solving, cognitive restructuring, and relaxation, through discussion, role-play, and mutual support to enhance emotional processing, coping skills, and relationship health [16]. Trials of LIFT found that participants involved in the coping portion of the intervention had reductions in trauma related stress and avoidance coping compared to the control [16, 17].

An adaptation of LIFT, ImpACT, shortened LIFT to 6 sessions of group and individual treatment, and it was culturally adapted for use with South African women with sexual violence histories. The pilot test of the intervention demonstrated clinically significant reductions in PTSD symptoms as well as decreased use of avoidant coping at 3-month follow-up [18]. Attendance at the in-person intervention was low, with 4 individual sessions attended by 65.6% of participants and only 25% attending any of the 2 group sessions, which may have attenuated the impact of the intervention. Researchers hypothesized that privacy issues or stigma around attending group sessions may have been a factor inhibiting attendance. These limitations underscore the need for a more accessible delivery system, such as mobile health (mhealth), that facilitates privacy and can be accessed as needed. In general, given the high prevalence of violence and mental health consequences of violence, there is a dearth of interventions [19] to address mental health in PWH [20].

This paper examines the outcomes of a pilot trial of an mHealth adaptation of LIFT. Interest in health interventions delivered through mobile devices has grown over the last decade with the rapid increase in mobile cellular coverage and use [21]. Mobile health and ecological momentary interventions have shown small to medium effect sizes in addressing depression, anxiety, stress, and positive psychological symptoms [22]. Internet/mobile PTSD interventions show variable impact. A Cochrane review cited that quality of the evidence was low for ‘internet-based’ PTSD interventions but concluded there is some evidence of efficacy [23]. A randomized controlled trial (RCT) of My PTSD Coach showed improvements in PTSD symptoms, depression, and psychosocial functioning at posttreatment compared to a wait list, although no group differences were identified [24].

While mHealth interventions may have smaller effect sizes, from a public health perspective, their greater accessibility, lower cost, and potential for real-time support make them promising tools for broader implementation. A particularly innovative mHealth approach is the Just-In-Time Adaptive Intervention (JITAI) model, which delivers support at the moment it is most needed, based on real-time data such as stress levels, user input, or behavior patterns. JITAIs aim to enhance user engagement and increase intervention effectiveness by tailoring prompts, feedback, or coping tools to users’ current states or contexts [25]. JITAI is relatively new to the HIV field. One pilot found that a mobile intervention intended to boost positive mood in JITAI format was feasible and acceptable among 22 sexual minority men living with HIV, though findings suggest further adaptation is needed to improve usability and tailoring [26]. Despite limited application in HIV-specific contexts, a growing body of evidence from other health domains demonstrates the promise of JITAIs: multiple meta-analyses and systematic reviews have reported medium to large effect sizes across a range of health behaviors and psychological outcomes [27, 28]. The NOLA Gem app, developed for this pilot study is a trauma-informed, coping-focused JITAI designed to support users in real-time as they navigate daily stressors.

Many mHealth interventions for PWH in the US have focused on medication adherence or treatment adherence using SMS text reminders [29]. A recent systematic review of mHealth interventions aimed at improving quality of life among PWH found that most studies were conducted in Asia, with none specifically addressing HIV-related quality of life in the United States [30]. Even fewer studies aim to address mental health and coping in PWH. Two separate interventions have used the Cognitive Behavioral Stress Management (CBSM) model developed by Antoni et al. [31] specifically for people with HIV to provide psychoeducation related to stress, relaxation, and coping skills. The run4love intervention in China consisted of an adapted CBSM course using articles and audio content sent over WeChat 3 to 5 times weekly [32]. Randomized clinical trials of run4love found significant reductions in depression symptoms for participants compared to controls [33]. However, none of these apps incorporated elements of JITAI. To our knowledge, no studies in the U.S. have examined outcomes from an mHealth app specifically designed to address mental health and coping more broadly among PWH while incorporating elements of JITAI. In general, given the high prevalence of violence and mental health consequences of violence, there is a dearth of interventions [19] to address mental health in PWH [20].

This study was conducted in the New Orleans metropolitan area, Louisiana. In addition to having higher-than-average rates of HIV [34, 35], this region is also marked by high rates of poverty, health disparities [36], violence [37], and childhood adversity [38], factors that compound the mental health burden for PWH. Natural disasters such as Hurricane Katrina have also left lasting impacts on the mental health in the region [39]. Given the potential value of a trauma-informed, coping-focused mHealth intervention for this population, the primary aim of this pilot study was to conduct preliminary analyses to compare outcomes (coping, PTSD symptoms, emotion regulation, and stress) over time between participants in the pilot trial of *NOLA GEM* and a control group across a 3-week (21-day) intervention period. Other studies have utilized positive early outcomes from pilots to inform full scale efficacy trials [40, 41]. These exploratory findings offer early insight into potential intervention-related patterns, including directionality and differences in level of completion, to help motivate and refine the design of subsequent large-scale evaluations.

## Methods

### Study Design

This quasi-experimental pilot study examined preliminary outcomes of the NOLA Gem app, a psychoeducation app designed to improve coping skills in PWH. The goals of this analysis were to obtain preliminary information about directionality and differences between level of completion rather than to assess causality. NOLA Gem was adapted from LIFT, an evidence-based intervention to address trauma symptoms and sexual risk behavior in people with HIV and child sexual abuse histories [16, 17]. Participants were randomly assigned 1:1 control/intervention until total participants reached 16, after which participants were assigned solely to intervention, resulting in an end *N* = 24 for intervention and *N* = 8 for control. This decision was made due to challenges with clinic closures associated with Covid-19 and Hurricane Ida that impacted recruitment and allowed us to increase the sample size for the intervention group, incentivize usage of the app, and to better assess acceptability and feasibility. Furthermore, seeing the benefits from the app reported by intervention group participants, we did not want to withhold intervention from anyone. The intervention group was given access to all the modules and associated skills for NOLA Gem. While both groups completed daily diaries to assess a variety of daily experiences and symptoms, for the intervention group, specific survey responses also triggered a notification from the app suggesting that the participant complete certain skills (for example, reporting significant stress). All of the modules and skills in the app were also available to intervention participants to access at any time during the intervention period. An overview of the intervention content and design as well as specific app triggers are discussed at more length elsewhere [42]. Controls were not given access to the app modules and instead only had a resources page for local and national services such as counseling.

Recruitment took place in New Orleans, LA through convenience sampling by: (1) reaching out to eligible participants taking part in a larger longitudinal cohort of PWH, the New Orleans Alcohol Use in HIV (NOAH) study [43], (2) distributing flyers to 12 different organizations and clinics working with PWH in the Greater New Orleans area. Study enrollment took place between October 2023-August 2024. The following eligibility criteria had to be met for participation: (1) 18 years of age or older, (2) self-reported to be on antiretroviral therapy (ART), (3) smartphone ownership, and d) participant indicated experiencing at least one incidence of violence on the screener, which covered a variety of potentially traumatic experiences. Approval for this study was granted by [Redacted] social and behavioral institutional review board. An oral consent form was read to each participant and verbal consent was recorded in REDCap [44]. Participants were reimbursed for their time and effort, including a $30 Amazon gift card for initial baseline, $20 Clincard immediate post-assessment and 30-day follow up, respectively, and a $10 Walmart gift card for the 90-day follow up. Participants earned $2 for each daily diary survey completed.Once treatment-arm enrollment, exclusively, was rolled out, participants were paid $5 via ClinCard for each session completed (described below). An additional $15 bonus was provided if all sessions were completed.

### Intervention

The NOLA GEM app contained the following psychoeducation sessions based on the LIFT intervention: 1) Breathwork (skill: practicing belly breathing for relaxation, 2) Journaling (skill: drawing or writing for 10–15 min), 3) Understanding Coping (skill: learning about types of coping), 4) Coping Strategies (skill: learning to choose and build multiple adaptive coping strategies), 5) Goal setting (skill: learning how to break down problems into achievable goals), 6) Challenging negative thoughts (skill: understanding types of negative thoughts and how to challenge them), and 7) Mindfulness (skill: practicing mindfulness in everyday life with a guided meditation). These psychoeducation modules and associated skills were available to the intervention participants at all times, however suggestions for skills practice were triggered by specific daily diary responses as part of a JITAI design. For example, reporting high stress would trigger a suggestion for breathwork. A fuller description of the app’s JITAI triggers has been previously described [42].

### Data Collection

SMS text links to daily diaries were issued twice per day at 9:00 AM and 6:30 PM for baseline and control for 3 weeks through the NOLA Gem app. The surveys took approximately 5 min to complete. Participants were instructed to complete each survey within a three-hour window from when the text was sent. Data were extracted from the NOLA Gem app, processed, and indices were calculated.

### Measures

*Baseline variables* included gender, age, race, education, income, and digital self-confidence. Gender options included male, female, non-binary, and a “prefer to self-describe” open-ended response option. Race categories matched those in the US census, however only Black, White, Mixed Race, and other were selected by participants. Education and income were measured as in the NOAH study [43]. As our sample size was small, a few selected variables were entered into the model and race was collapsed given low variability. Since the majority of participants identified as Black or African American, race was operationalized in regression as a binary variable of Black/African American vs. non- Black/African American. We used the digital self confidence [45] score to access readiness and ability to use digital technology. The score was calculated as the sum of four items, each rated on a 4-point Likert scale ranging from *strongly agree* to *disagree*: (1) “I use a digital device frequently,” (2) “Most of my friends use digital devices,” (3) “I can usually get help if I am stuck,” and (4) “I feel confident using most digital devices.”

Additionally, some baseline variables were used to assess initial mental health related differences between intervention and control. These included:

*Anxiety and depression* were measured using the Hospital Anxiety and Depression Scale (HADS), a validated and reliable screening tool consisting of 14 items (including 7-item subscales for anxiety and depression) [46].

*Alcohol consumption* was measured through the Alcohol Use Disorders Identification Test brief screener (AUDIT-C) consisting of three questions covering frequency of drinking and binge drinking [47]. Questions were summed to receive a total possible range of 0–12.

All regression outcomes used daily diary measures. The following outcomes were assessed:

*PTSD*: Items assessing PTSD symptoms were adapted from the PCL-5, a well-validated self-report measure of PTSD symptoms [48] and modified for daily responses. Selected PCL-5 items have been used with demonstrated internal consistency in previous ecological momentary assessment studies [49] and showed excellent internal consistency for this study (Cronbach’s alpha = 0.89). PTSD symptoms were assessed twice per day. Response sets for PTSD were on a 1 to 5 scale. PTSD symptomatology was operationalized as a binary variable where 1 = endorsing any level of the following: (1) “How much were you bothered by disturbing thoughts, activities, or feelings about difficult life events that have happened to you?”, (2) “How much were you bothered by avoiding thoughts, activities, or feelings about difficult life events that have happened to you?”, (3) “How much were you bothered by feeling distant or cut off from other people and/or feeling emotionally numb?”, (4) “How much were you bothered by difficulty concentrating, feeling jumpy or easily startled, feeling overtly alert, or feeling irritable or angry?” and (5) “How much were you bothered by having strong negative beliefs about yourself, other people, or the world (for example, having thoughts such as: I am bad, there is something seriously wrong with me, no one can be trusted, the world is completely dangerous)?”

*Coping* was measured using an adapted version of brief COPE developed by Carver [50] and comprised of 14 subscales, with 2 items on each scale. Though the factor structure is debated, a systematic review found Brief COPE is commonly modified and that a 2 factor structure comprising of adaptive/active coping vs. maladaptive/avoidant coping is supported [51]. We used a checklist of selected items and divided items into:

*Maladaptive Coping*: A maladaptive coping indicator was dummy-coded 1 one where participants endorsed at least one of the following in response to a stressful event: (1) refusing to believe that it has happened, (2) using alcohol or other drugs to help me get through it, (3) criticizing myself, (4) blaming myself for things that happened, and (5) expressing my negative feelings.

*Adaptive Coping*: An adaptive coping indicator was dummy-coded 1 where participants endorsed at least one of the following in response to a stressful event: (1) trying to come up with a strategy about what to do, (2) looking for something good in what is happening, (3) doing something to think about it less, (4) accepting reality of the fact that is has happened, (5) expressing my negative feelings, (6) trying to find comfort in my religion or spiritual beliefs, 8) trying to get advice or help from other people about what to do, and 9) concentrating my efforts on doing something about the situation I’m in.

*Stress*: Participants were asked: “How much stress are you feeling right now?” Those who answered some or a lot were coded as one while those who answered none were coded as 0.

*Stress Control*: Participants were asked how much in control they felt about a stressful event they experienced in the day. Those who answered somewhat or very much were coded as one while those who responded not at all were coded 0.

*Rumination*: This question was from a study that surveyed 10 different emotional regulation strategies, one of which was rumination [52]. Participants were asked: “How often did you use the following strategies to deal with stress today?” I thought over and over again about my emotions. Those who answered some or a lot were coded as one while those who responded no at all were coded as 0.

*Alcohol Intake*: Participants were asked: “Since last night/your last diary entry, how many drinks containing alcohol have you had?” Those who answered one or more were coded as one while those who had no drinks were coded as 0.

For regression, an additional variable was created based on whether the user completed a higher number of modules than the median (high user) or lower than median/equal to the median (low user). This was operationalized into a higher user vs. low user/control variable.

### Data Analysis

First, descriptive statistics were computed for both the intervention and control groups, utilizing t-tests or Mann-Whitney U for continuous variables and chi-square or Fisher’s exact tests for categorical, based on cell sizes. To improve balance, inverse probability of treatment weighting (IPTW) was used to estimate the average treatment effect (ATE). These were created using a propensity score (PS) generated from sex, age, digital self-confidence, education, income, and race. Contributions of participants were weighted by 1/PS for intervention and 1/(1 − PS) for controls. Intra-Class Correlation Coefficients (ICC) were run to examine variance. ICCs ranged from 0.79 to 0.89, indicating that a substantial portion of variance in the outcome was attributable to between-participant differences rather than within-person fluctuations over time and justifying the use of random intercept models with Participant ID as a grouping factor. A series of random intercept regression models were conducted to account for within-subject clustering. Variables were added in succession to assess the impact of each variable on the model. Subsequently, we estimated the same models using Bayesian random intercept logistic regression. Bayesian models have been documented to be beneficial and have stabilizing effects in cases of logistic regression in small sample sizes [53] and have been suggested to be useful in the context of pilots [54]. Models accounted for clustering within participants over time, with predictors added sequentially to assess their influence. To improve posterior stability and prevent inflated coefficient estimates common in small-sample or separated logistic models, we standardized continuous predictors and used weakly informative Normal priors for regression coefficients (normal(0, 1)). This Bayesian approach was selected based on simulation evidence and applied research showing that normal priors offer improved regularization and convergence compared to heavier-tailed alternatives like Cauchy or Student’s t in sparse-data contexts [55].

## Results

Thirty-two participants were enrolled and 30 participants completed as least one daily diary. Participants completed between three and 42 surveys (the maximum possible number) with an average of 28 surveys (67% of expected) completed during the study period. When comparing intervention and control groups, there were an average of 29 surveys completed for control and 27.6 completed for intervention with medians of 32 and 31, respectively.

Characteristics of participants at baseline are presented in Table 1. Gender showed the largest differences between intervention and control as 72.7% of the intervention group were men compared to only 25.0% in the control group, this difference was marginally non-significant (*p* = 0.055). The intervention group was also younger on average (Mean = 51.8 years, SD = 12.6) than the control group (Mean = 60.9 years, SD = 6.06), though this difference was not statistically significant (*p* = 0.176). Most participants were Black/African American (73.3%), and there were no significant racial distribution differences between groups (*p* = 0.931). Educational attainment was similar across groups (*p* = 0.918), with 50.0% of participants reporting some college or higher, while 16.7% had less than a high school education. Digital self-confidence scores were slightly higher in the control group (Mean = 7.13, SD = 1.25) compared to the treated group (Mean = 6.14, SD = 2.19), but this difference was not statistically significant (*p* = 0.494). Income distribution was also comparable between groups (*p* = 0.680), with 43.3% of participants earning between $10,000 and $19,999 annually and 23.3% earning less than $10,000. Most participants in both groups fell into the “No Anxiety” range (treated = 63.6%, control = 87.5%). Borderline anxiety was more common in the treated group (27.3% vs. 0%), while the proportion meeting criteria for anxiety was similar (treated = 9.1%, control = 12.5%). The treated group had a higher average anxiety score (Mean = 7.23, SD = 4.01) compared to the control group (Mean = 5.00, SD = 4.07), but there were no significant differences for anxiety either as a summary score or categorical variable. The majority of participants also did not meet criteria for depression (treated = 77.3%, control = 87.5%). Borderline depression was rare (3.3% overall). Rates of depression were slightly higher in the treated group (18.2% vs. 12.5%) with slightly higher HADS depression summary scores (Mean = 4.82, SD = 4.16) compared to the control (Mean = 3.38, SD = 4.31), but neither of these were statistically significant. AUDIT scores were similar between groups (treated = 3.83, SD = 2.31; control = 3.25, SD = 2.22) with no significant difference (*p* = 0.898).

**Table 1:** Baseline characteristics of NOLA gem participants enrolled in intervention or control

	Intervention	Control	Overall	*P*-value
	(*N* = 22)	(*N* = 8)	(*N* = 30)	
**Gender**				
Male	16 (72.7%)	2 (25.0%)	18 (60.0%)	0.0578
Female	6 (27.3%)	6 (75.0%)	12 (40.0%)	
**Age**				
Mean (SD)	51.8 (12.6)	60.9 (6.06)	54.2 (11.9)	0.176
Median [Min, Max]	52.5 [28.0, 74.0]	61.0 [50.0, 72.0]	58.5 [28.0, 74.0]	
**Race**				
White	4 (18.2%)	0 (0%)	4 (13.3%)	0.931
Black	15 (68.2%)	7 (87.5%)	22 (73.3%)	
Mixed Race	2 (9.1%)	1 (12.5%)	3 (10.0%)	
Other	1 (4.5%)	0 (0%)	1 (3.3%)	
**Education**				
Less than HS	3 (13.6%)	2 (25.0%)	5 (16.7%)	0.918
High School	7 (31.8%)	3 (37.5%)	10 (33.3%)	
Some college or more	12 (54.5%)	3 (37.5%)	15 (50.0%)	
**Digital self confidence**				
Mean (SD)	6.14 (2.19)	7.13 (1.25)	6.40 (2.01)	0.494
Median [Min, Max]	5.50 [4.00, 10.0]	7.00 [5.00, 9.00]	6.50 [4.00, 10.0]	
**Income**				
Less than $10,000	5 (22.7%)	2 (25.0%)	7 (23.3%)	0.68
$10,000 to $19,999	8 (36.4%)	5 (62.5%)	13 (43.3%)	
$20,000 to $75,000	9 (40.9%)	1 (12.5%)	10 (33.3%)	
**HADS anxiety summed score**				
Mean (SD)	7.23 (4.01)	5.00 (4.07)	6.63 (4.08)	0.418
Median [Min, Max]	7.00 [1.00, 17.0]	4.50 [0, 13.0]	7.00 [0, 17.0]	
**HADS anxiety categories**				
No Anxiety	14 (63.6%)	7 (87.5%)	21 (70.0%)	0.601
Borderline	6 (27.3%)	0 (0%)	6 (20.0%)	
Anxiety	2 (9.1%)	1 (12.5%)	3 (10.0%)	
**HADS depression summed score**				
Mean (SD)	4.82 (4.16)	3.38 (4.31)	4.43 (4.17)	0.707
Median [Min, Max]	4.50 [0, 13.0]	1.00 [0, 12.0]	3.50 [0, 13.0]	
**HADS depression categories**				
No Depression	17 (77.3%)	7 (87.5%)	24 (80.0%)	1.00
Borderline	1 (4.5%)	0 (0%)	1 (3.3%)	
Depression	4 (18.2%)	1 (12.5%)	5 (16.7%)	
**AUDIT sum**				
Mean (SD)	3.83 (2.31)	3.25 (2.22)	3.73 (2.25)	0.898
Median [Min, Max]	3.00 [1.00, 9.00]	3.00 [1.00, 6.00]	3.00 [1.00, 9.00]	
Missing	4 (18.2%)	4 (50.0%)	8 (26.7%)	

Table 2 presents regression results comparing intervention and control arms. Self-reported alcohol intake was marginally higher in the intervention group. Both maladaptive coping and drinking were also marginally higher among participants who identified as Black or African American, though these differences were not statistically significant. PTSD was significantly lower for all participants across groups at week 2 (OR = 0.53; 95% CI: 0.31–0.91). Stress was lower across all groups at both week 2 (OR = 0.58; 95% CI: 0.33–0.96) and week 3 (OR = 0.47; 95% CI: 0.26–0.81). At week 2, intervention participants had 50% lower odds of maladaptive coping compared to controls, though this effect was marginally non-significant (OR = 0.50; 95% CI: 0.24–1.06). They also had significantly higher odds of feeling in control of their stress (OR = 3.59; 95% CI: 1.40–8.84).

**Table 2:** Regression results for daily diary outcomes between NOLA gem users (intervention) and control

	Maladaptive coping	Adaptive coping	PTSD	Rumination	Stress	Stress control	Alcohol intake
Week 2	0.99 (0.53–1.77)	0.81 (0.52 − 1.24)	**0.53** **(0.31–0.91)**	1.04 (0.52–2.01)	**0.58** **(0.33–0.96)**	0.73 (0.34–1.55)	1.05 (0.52–2.03)
Week 3	1.17 (0.68–2.09)	0.85 (0.54–1.29)	1.06 (0.63–1.81)	1.34 (0.69–2.71)	0.47 (0.26–0.81)	0.96 (0.45–2.12)	0.84 (0.46–1.53)
Treatment	1.13 (0.30–4.53)	1.03 (0.41–2.55)	0.71 (0.23–2.22)	1.34 (0.41–4.63)	1.21 (0.27–5.11)	0.79 (0.20–2.94)	1.87 (0.59–6.32)
Race	3.23 (0.74–12.72)	0.52 (0.24–1.14)	1.33 (0.51–3.91)	1.66 (0.50–4.71)	0.60 (0.17–2.47)	0.65 (0.17–2.70)	1.98 (0.56–6.53)
Sex	0.42 (0.13–1.54)	1.08 (0.48–2.32)	0.66 (0.22–1.97)	0.82 (0.27–2.63)	1.09 (0.30–3.42)	2.36 (0.67–8.49)	0.46 (0.15–1.35)
Age	0.97 (0.91–1.03)	0.99 (0.96–1.03)	1.03 (0.98 − 1.08)	1.05 (0.99–1.10)	0.95 (0.88–1.01)	1.02 (0.94–1.09)	1.04 (0.98–1.11)
Baseline	1.26 (1.12–1.47)	1.06 (1.02–1.11)	1.01 (1.01–1.02)	1.06 (1.04–1.07)	1.05 (1.04–1.07)	1.03 (1.01–1.05)	1.07 (1.04–1.10)
Week2*intervention	0.51 (0.24–1.14)	0.68 (0.39–1.17)	0.99 (0.49–2.12)	0.52 (0.20–1.26)	1.56 (0.80–3.16)	**3.59** **(1.40–8.84)**	1.10 (0.52–2.42)
Week3*intervention	**0.33** **(0.14–0.73)**	0.69 (0.38–1.21)	**0.45** **(0.22–0.92)**	**0.27** **(0.11–0.68)**	0.98 (0.46–2.09)	**4.82** **(1.82–12.96)**	0.64 (0.28–1.28)

Results are presented in odds ratios and 95% confidence intervals

*Bolded items indicate significance

Most intervention effects emerged at week 3. Intervention participants had 67% lower odds of maladaptive coping (OR = 0.33; 95% CI: 0.14–0.73), 55% lower odds of PTSD (OR = 0.45; 95% CI: 0.22–0.92), and 73% lower odds of rumination (OR = 0.27; 95% CI: 0.11–0.68) compared to controls. Similar to week 2, feelings of control over stress increased (OR = 4.82; 95% CI: 1.82–12.96).

From week 1 to week 3, the odds of maladaptive coping in the intervention group decreased by 61%, while increasing by 17% in the control group. The odds of PTSD decreased by 52% in the intervention group, compared to a 6% increase in the control group. The odds of rumination decreased by 64% in the intervention group, while increasing by 34% in the control group. Finally, the odds of stress control increased by 362% in the intervention group, compared to a 4% decrease in the control group.

Table 3 displays the results comparing high completion and low completion/control groups. Maladaptive coping and drinking were marginally higher among high completers and participants who identified as Black or African American, though these differences were not statistically significant. PTSD (OR = 0.41; 95% CI: 0.26–0.65) and stress (OR = 0.63; 95% CI: 0.40–0.98) significantly decreased across both groups at week 2, but not at week 3. At week 2, high completers reported significantly higher PTSD symptomatology (OR = 2.37; 95% CI: 1.08–5.21) and greater stress control (OR = 3.41; 95% CI: 1.30–9.57) compared to low completers and controls.

**Table 3:** Regression results for daily diary outcomes between NOLA gem high completers and low completers/control

	Maladaptive coping	Adaptive coping	PTSD	Rumination	Stress	Stress control	Alcohol intake
Week 2	0.96 (0.55–1.59)	0.71 (0.49–1.02)	0.41 (0.26–0.65)	0.74 (0.41–1.33)	**0.63** **(0.40–0.98)**	0.96 (0.56–1.74)	1.11 (0.67–1.75)
Week 3	1.15 (0.69–1.93)	0.78 (0.54–1.13)	0.82 (0.54–1.27)	1.18 (0.66–2.16)	0.70 (0.43–1.14)	1.22 (0.65–2.25)	0.87 (0.54–1.37)
Treatment	2.53 (0.76–8.35)	1.39 (0.64–3.01)	0.87 (0.26–2.89)	1.23 (0.39–3.60)	0.57 (0.15–2.08)	0.67 (0.20–2.46)	1.59 (0.50–4.51)
Race	3.13 (0.76–12.69)	0.50 (0.22–1.13)	1.40 (0.47–4.10)	1.77 (0.60–5.63)	0.66 (0.19–2.34)	0.63 (0.16–2.57)	1.84 (0.53–6.10)
Sex	0.44 (0.14–1.56)	1.12 (0.55–2.37)	0.79 (0.26–2.45)	0.86 (0.31–2.47)	1.05 (0.31–3.13)	2.26 (0.57–8.26)	0.39 (0.13–1.17)
Age	0.97 (0.91–1.04)	0.99 (0.96–1.03)	1.03 (0.98–1.09)	1.06 (1.00–1.11)	0.95 (0.88–1.00)	1.02 (0.95–1.08)	1.04 (0.98–1.11)
Baseline	1.23 (1.09–1.43)	1.06 (1.02–1.10)	1.01 (1.01–1.02)	1.06 (1.04–1.08)	1.06 (1.04–1.08)	1.03 (1.01–1.04)	1.07 (1.05–1.10)
Week2*intervention	0.50 (0.24–1.06)	0.78 (0.42–1.41)	**2.37** **(1.08–5.21)**	0.92 (0.36–2.72)	1.62 (0.72–3.65)	**3.41** **(1.30–9.57)**	1.10 (0.51–2.35)
Week3*intervention	**0.31** **(0.13–0.72)**	0.72 (0.38–1.33)	0.61 (0.26–1.39)	**0.14** **(0.04–0.40)**	**0.14** **(0.04–0.41)**	**8.30** **(2.69–25.91)**	**0.27** **(0.11–0.67)**

Results are presented in odds ratios and 95% confidence intervals

*Bolded Items Indicate significance

Most intervention effects were observed at Week 3. High completion participants had 69% lower odds of maladaptive coping (OR = 0.31; 95% CI: 0.13–0.72), 86% lower odds of rumination (OR = 0.14; 95% CI: 0.04–0.40), 86% lower odds of reporting stress (OR = 0.14; 95% CI: 0.04–0.41), and 73% lower odds of drinking (OR = 0.27; 95% CI: 0.11–0.67) compared to low completers and controls. In contrast, the odds of stress control were 8.3 times higher in the intervention group than in the low completers and control groups (OR = 8.30; 95% CI: 2.69–25.91). Though the odds ratio for PTSD in week 3 suggested a greater decrease in intervention compared to control, this was not significant.

From week 1 to week 3, the odds of maladaptive coping in the intervention group decreased by 54%, while increasing by 18% in the control group. The odds of stress decreased by 86% in the intervention group, compared to a 30% decrease in the control group. The odds of reporting stress control increased approximately sevenfold in the intervention group, while increasing by 22% in the control group. The odds of reporting alcohol intake decreased by 73% in the intervention group, while increasing by 7% in the control group. Finally, the odds of rumination decreased by 84% in the intervention group, compared to an 18% increase in the control group.

## Discussion

In this study, we examined the results of a preliminary, small sample, pilot trial of NOLA Gem, a mobile app designed to support mental health and coping among people living with HIV through JITAI components. Though no causal interpretations can be made from a sample this small, preliminary information about directionality and differences between level of completion can demonstrate the benefit of conducting future at scale studies. From week 1 to week 3, participants in the intervention group experienced significant reductions in maladaptive coping, rumination, and PTSD symptoms compared to the control group, along with increased feelings of control over stress at both timepoints. These effects were especially pronounced among high completers, who demonstrated greater improvements in maladaptive coping, rumination, and stress control compared to both low completers and controls. High completers also showed significant decreases in overall stress and drinking. Interestingly, PTSD symptoms increased in the high completion group at Week 2 but declined by Week 3.

Coping was a central focus of the intervention’s design, reflecting the adaption of LIFT, with several modules aimed at helping participants recognize coping strategies that may be harmful to their mental and physical health. Results showed that maladaptive coping significantly decreased in the intervention group compared to the control group, while adaptive coping did not differ significantly between groups. Most interventions examining coping skills take place over months [56–58], so it is possible that participants began reducing maladaptive behaviors after learning about their negative consequences but had not yet had enough time or practice to effectively implement new adaptive coping strategies.

Two studies that examined the sequence in which coping skills were taught found that distress reactions to stressful thoughts were most reduced when participants learned about the negative impacts of avoidance coping before learning about acceptance strategies [59, 60]. These studies also found lower adherence to acceptance-based coping techniques when they were introduced first, and higher adherence in the following phase. This may suggest that maladaptive coping strategies are more easily understood and recognized, and that introducing them first in psychoeducation interventions could facilitate a better understanding of adaptive strategies. It also suggests that adaptive strategies may require more time and practice to be effectively used. In addition, a large body of literature has consistently found that maladaptive coping is a stronger predictor of mental health conditions such as anxiety, depression, and borderline personality disorder [61, 62]. The reasons for this are debated. Some evidence suggests that the effectiveness of adaptive strategies depends heavily on contextual factors such as emotional intensity or the type of emotion being regulated. For example, Aldao and Nolen-Hoeksema [63] found that individuals who used a greater variety of adaptive strategies, rather than simply using them more often, were less likely to experience mental health problems, underscoring the importance of coping flexibility. In line with this, the NOLA Gem intervention explicitly targeted maladaptive coping behaviors and aimed to increase users’ access to a broader set of adaptive strategies in real-time. These features may help reduce mental health symptoms by promoting more flexible and contextually appropriate coping.

In both the intervention and control groups, participants experienced a reduction in stress levels; however, the decrease was not significantly greater in the intervention group. This suggests that the act of completing daily diaries may have contributed to stress monitoring and evaluation, similar to the benefits observed with journaling [64, 65]. While limited studies have examined the impact of digital emotion tracking on mental health, some have reported increased reporting of positive emotions, potentially due to enhanced recall of emotional experiences [66, 67]. However, feelings of control over daily stressful events increased more in the intervention group compared to the control group. Previous research has found that adaptive coping strategies can enhance perceived control over stress [68–70], which in turn mediates and moderates the relationship between stress and mental well-being [68, 69, 71, 72]. This suggests that the intervention group may have modified the participant’s overall perceived capacity for stress management, a key component of coping self-efficacy, even when overall stress levels remained comparable between groups. As not all experiences of stressful events are able to be avoided, stress control management may be particularly important for overall mental health.

Reported PTSD symptoms significantly decreased by week 3 in intervention compared to control. However, high completers showed a different pattern. Their PTSD symptoms increased in week 2 before decreasing in week 3 (non-significantly). For both overall intervention and high completers, repetitive thinking only significantly decreased in week 3, showing a delayed effect. Some research has indicated that symptoms of PTSD can be initially exacerbated during emotion and coping interventions, particularly meditation. For example, a study of veterans undergoing mindfulness meditation training found significant increases from control for the first 3 weeks of intervention followed by a significant reduction [73]. Another study found that active PTSD symptoms increased participants’ likelihood of experiencing distress during mindfulness meditation, suggesting that reflecting on one’s emotions and thoughts can trigger memories and feelings related to trauma [74]. This may explain why the reduction in PTSD symptomology and repetitive thinking was delayed in participants. This also suggests that meditation and breathing components may be introduced more slowly, with a greater focus on trauma informed practices including emphasizing performing skills in a safe and comfortable environment and education that these exercises may trigger initial discomfort. Additionally, future interventions may need to extend beyond this three-week period to bolster impact.

Models comparing those that engaged with the app more on average (high completers) with those with low engagement and controls found self-reported alcohol use was significantly lower in high completers in week 3 and a larger effect for most outcomes in contrast to models that simply compared intervention and control. This result is not surprising as much research has documented the importance of engagement in outcomes. Both a systematic review of digital behavior change interventions [75] and a more recent app for men with HIV [76] found that the desired behavior change was closely tied to user engagement. Heavy alcohol use can be thought of as a downstream effect of maladaptive coping or a maladaptive coping technique itself, particularly when the individual uses alcohol or other substances to dull and repress uncomfortable emotions [5, 77]. Given our results, higher engagement with the app may be necessary to drive more specific behavior changes beyond general emotional outcomes, particularly to reinforce psychoeducation on maladaptive coping and support the development of alternative emotion regulation strategies.

### Optimization and Implementation Considerations

While these findings suggest a promising, if highly preliminary, efficacy profile for NOLA Gem, broader questions of acceptability and usability (proximally) and sustainable clinical workflow integration (distally), remain. The complexity and nontrivial user interaction costs associated with the JITAI design render these questions more urgent [78, 79]. The 94% response rate of ≥ 1 daily diary demonstrates crude feasibility, in accord with our preregistered benchmark [42]. But in-progress mixed methods acceptability and usability analyses show that, while many NOLA Gem users expressed appreciation of the skills-building prompts, even intuiting the underlying “just-in-time” mechanism of action, the twice-daily extended EMA inputs remain burdensome [80]. Future directions to address these frictions include micro-randomized controlled trials, undertaken to isolate contextual drivers of successful skills prompt → proximal outcome pathways, and reinforcement learning integration, continually optimizing individualized JITAI decision rules [81, 82]. In either case, daily diary measures that are mis-attuned to the daily stressors of PWH users could be pruned for efficiency’s sake. Taking personalization potential to the next step, dynamic content creation via properly aligned LLM, even tapping a vectorized LIFT knowledge backend, can complement adjacent future optimizations by providing more adaptive, context-aware, and natural language-based interactions [83, 84].

The healthcare technologies non-adoption, abandonment, scale-up, spread, and sustainability framework implicates complexity, mutually exacerbated across the intervention, the value proposition, and the implementation setting, as the foremost barrier to health tech “mainstreaming” [85]. NOLA Gem optimization must, in short, prioritize simplicity in onboarding, navigation, and user experience, to lay the groundwork for future clinical integration and sustainment. EMA is not, necessarily, a barrier to successful clinical workflow integration and cross-site scaling. PositiveLinks, a clinic-based intervention combining a smartphone app, patient dashboard, and administrator panel, was recently inducted into the CDC’s *Compendium of Evidence-Based Interventions and Best Practices for HIV Prevention*, features “daily check-ins” [86, 87]. These EMA-like prompts include measures of mood and stress, pointing toward the potential viability of NOLA Gem within established outpatient workflows.

### Limitations

This study has several limitations that should be considered when interpreting the findings. First, allocation to intervention and control conditions was only partially randomized. While random assignment was used until 16 participants were enrolled, subsequent participants were assigned to intervention only. This may have introduced selection bias, particularly if individuals with different characteristics joined after the initial randomization, leading to imbalances between groups. In this case, the control had no White participants and was majority female, while intervention was majority male. These large differences in a small sample can make estimation with matching or weighting more difficult. Although IPTW was used to estimate the ATE, visual inspection of the distribution of weights indicated only partial overlap between the intervention and control groups. Limited overlap reduces the ability to make strong causal claims and may affect the generalizability of the findings.

Second, the intervention and data collection period lasted only three weeks. This limited timeframe may not have been sufficient to observe long-term changes, particularly in adaptive coping behaviors, which often require more time and practice to implement effectively [70]. Future studies should include longer follow-up periods to assess the durability of intervention effects. Third, as with all self-report studies, results are subject to social desirability bias. Participants may have underreported behaviors perceived as negative. For example, after learning more about what maladaptive coping is, participants may have become more reluctant to report such behaviors due to stigma or feelings of shame. Finally, the relatively small sample size may have limited the power to detect subtle effects or interactions over time. In logistic regression models, small sample sizes, especially with sparse events or clustered data, can lead to inflated or unstable effect estimates, emphasizing the need for cautious interpretation. ICC values indicated that a substantial portion of the variance in outcomes was attributable to between-person differences, reinforcing the need for multilevel modeling. Though daily diary completion rate was low for some participants, average completion was 67%, a rate which is consistent with a recent metanalysis finding 75% completion rate in a pooled sample of over 19,000 individuals across studies [88]. Overall, results suggest that completion rate was acceptable, but replication with a larger, more diverse sample would improve estimate precision and strengthen the generalizability of these findings.

## Data Availability

Not applicable.
